# "The Hospital" Nursing Mirror

**Published:** 1901-03-09

**Authors:** 


"The Hospital" Nursing Mirror.

Being the Nursing Section of "The Hospital."

[Contributions for this Section of "The Hospital" should be addressed to the Editor, "The Hospital" Nursing Mirror, 28 & 29 Southampton Street Strand, London, W.C.]

### Notes on News from the Nursing World.

### HOW SISTER OWEN WAS DROWNED.

The death of Sister Owen, A.N.S.R., of No. 2 Stationary Hospital, Elandsfontein, by drowning, has already been announced in our columns. A correspondent in South Africa now sends a few details of the sad event which terminated so young and promising a life. On Friday, January 25th, Sisters Owen and Taylor, three officers and two civil surgeons went for a sail on a dam, two and a half miles out from Elandsfontein. All went well for a time till a sudden squall upset the boat and all its occupants. Both sisters were rescued each by a doctor and given a holding on the upturned boat. As no one seemed about and it was highly improbable that any person had witnessed the accident, one of the surgeons volunteered to swim to the side for assistance. As he looked back he saw that all were still safely holding on, but on reaching the bank to his dismay he observed that one had disappeared. It seems that a Zulu boy with a boat had seen the disaster and hastened to the rescue, but on getting near had come in violent contact with the upturned craft, and thus caused Sister Owen to loose her hold; she sank at once. The doctor, as soon as the others had landed and gone back to Elandsfontein, got into the boat and pulled out to the scene of the disaster. He waited about for half an hour, but with no results, and then swam about and under the boat for another hour to see if the body could be found, but without avail. Though dragging was resorted to, the body did not rise to the surface till the morning of the 29th. It was buried the same afternoon. The funeral was attended by all the sisters, officers and men of the R.A.M.C., also the civil surgeons, and some 200 to 300 men. Miss Owen, our correspondent adds, will be much missed. "She was young, bright, amiable, a devoted and capable nurse, and a general favourite with all."

### JOHANNESBURG HOSPITAL.

Miss Young and her staff have resumed their work at Johannesburg, and the military authorities are getting the sanitary arrangements put in order once more. It is, however, unlikely that the former residents will be allowed to return for several months, but when the war is really over, it is hoped that Johannesburg will, in a year or so, be more flourishing than ever, and the hospitals be again in working order. Ten or twelve years ago, the hospital work was done by a community of sisters, who laboured in a devoted manner, though the necessities of life were only to be obtained at abnormal prices, and even water was not to be had except on payment of a shilling for a bucketful! When a change was made, it took the committee some time to become accustomed to the requirements of modern-trained nurses.

### THE CHARGE OF HUSBAND HUNTING.

One of the daily papers includes among the attractions offered to nurses for pursuing their vocation in South Africa the prospect of obtaining a wealthy husband. To quote our contemporary: "If the nurses be young, in a land where the men are many and women so few, there should be an excellent prospect of marriage for those so inclined. The ordinary hospital doctor would be a small catch beside the wealthy English colonists who require nursing, and are sentimental in their new home." There is a suggestion here which cannot be too strongly repudiated. It is true that one or two of the nurses who went out to South Africa have since been married, but neither nurses in hospitals at home, nor nurses of the sick and wounded in distant lands, have foresworn matrimony. We have seen it stated that there is "a craze for marrying hospital nurses," and the author of the statement justified it on the ground that the training of the nurse—training which teaches her to use her intelligence in an emergency, to be methodical, and to keep the home healthy—renders her pre-eminently desirable as a wife. To this might easily be added many other recommendations, and we unhesitatingly assert that the capable nurse does, as a rule, make an admirable wife. But the idea that she is on the look-out to "catch" the ordinary hospital doctor, or might be tempted to go to South Africa merely in the hope of securing a rich husband, is exceedingly and gratuitously offensive. There are several false conceptions of the nursing vocation, but if once people became imbued with the notion that it included husband-hunting, the results would be disastrous. Happily the demeanour and the devotion of the overwhelming majority of nurses supply a refutation to a mischievous and ridiculous theory.

### MISS ROGERS AND THE EAST PRESTON GUARDIANS.

The East Preston Guardians having gained their point in reference to Miss Rogers, and the superintendent nurse having, in accordance with the request of the Local Government Board, resigned her post, an attempt is now being made to claim absolute authority over whoever may be her successor. One of the guardians thought well to adopt a blustering tone, and to intimate that unless the Local Government Board gave the Guardians power to suspend the superintendent, he would not sanction another appointment. His sanction will, doubtless, be dispensed with. The case of Miss Rogers has at least shown the importance of the Local Government Board refusing to increase the powers of the Guardians. The fact that another nurse at the East Preston Workhouse Infirmary has resigned, points to the conclusion that friction will not necessarily cease with the retirement of Miss Rogers. As the medical officer, in his evidence at the inquiry by the representatives of the Local Government Board, said that there had been "a decided improvement in the state of things at the infirmary since Miss Rogers had been in charge," and as even her most censorious critic on the Nursing Committee declared that "there was not the slightest complaint against her in her capacity as nurse," a useful career should be before her elsewhere.

### THE QUESTION OF SKIRTS.

One of the first things to strike a nurse taking up work in a Scottish district is the comparative absence of draggle-tails. Women work in their homes, or at the mills, or in the fields, in a short round "coat," *i.e., petticoat.* It may be that the Scotch possess neater feet and ankles than their English sisters, owing, perhaps, to the general habit of going barefoot in their early years; but however that may be, it is a pleasant sight to see the "guidwife" in the coarse but warm stockings of her own knitting, and the wooden shoes or "clogs" which keep her dry and comfortable in all weathers.

### NURSING IN BORDIGHERA.

ALTHOUGH there is only a branch of a nursing institution in Bordighera, there are five nursing sisters in the town. One of them, who for four years has nursed chiefly for one doctor, has lately made an experiment. The doctor having to go last month for change of air, and there being very little serious illness in the place, she started some courses of practical lessons in nursing, "How to make an invalid comfortable." It was not intended that the knowledge gained by the persons attending the courses should prevent people having trained nurses in cases of serious illness, but the idea was merely to help those who daily have to think of the comfort of the invalids, and are constantly with them.

### THE TRAINING AT BATH WORKHOUSE INFIRMARY.

THE Bath Board of Guardians have decided not to admit any more probationers for one year's training, and at a meeting the other day the following resolution was adopted:—"That in future, when nurses are required, probationers should be advertised for, who should after three months' trial, proving satisfactory, be bound for three years, to receive a salary of £5 for the first year, £10 for the second year, and £15 for the third year, with indoor uniform, board, lodging, and washing." One of the Guardians remarked that they had now a good nursing staff and they wished to keep it up to its present state of efficiency. Under the arrangement with the Affiliated Nursing Association they had the trouble of training the nurses who were then taken away from them before they had reaped any benefit from the tuition they had given. It is satisfactory that the Bath Guardians have recognised the wisdom of adopting the system of three years' training.

### A PROSPEROUS TRAINING INSTITUTION.

IT is noteworthy that, while Birmingham gives such scanty support to its District Nursing Society, the Birmingham Training Institution for Nurses is one of the most prosperous in the kingdom. The services rendered by the nurses attached to the institution are greatly appreciated by the private families who pay for them. Last year the receipts from this source amounted to no less than £3,183, and the number of families attended by the 75 nurses was 583. Out of its plenty too, the institution contributed, as usual, £150 to the District Nursing Society, and paid away £78 in pensions. The scale of salaries to the nurses was increased, and a large bonus was distributed among the staff.

### THE ACLAND HOME NURSES.

THE result of an exhaustive inquiry before the city coroner at Oxford into the circumstances of the death of Emma Eastbury is the complete vindication of the professional skill and care of Miss Mary Hall, a certificated nurse on the staff of the Sarah Acland Home. An attempt was made to attribute the death of Mrs. Eastbury, who was the wife of a pianoforte maker in the city, to an injection administered by Miss Hall. But against the evidence of several members of the family, the jury naturally set that of two medical men, one of whom said that he did not think the enema was unskilfully applied, or that the administration of it had anything to do with the patient's death; while the other, who, by the desire of the coroner, made a post-mortem examination, declared that death was almost absolutely, and certainly primarily, caused by lung trouble. In summing up, the coroner put the case very fairly. He said that in giving their verdict the jury would first determine what was the actual cause of death. They would also consider the importance of this institution to Oxford; they would consider the reputation and standing of the nurse; but neither of those facts should deter them if they thought it right to add to their verdict any expression of their opinion as to the treatment of the woman. They could leave that alone altogether in their discretion, or they could add a rider to their verdict and say she was properly treated or not skilfully treated, as they thought fit. The jury, thus enjoined, having intimated their verdict that "the cause of death was lung disease, and the use of the instrument did not accelerate the death in any way whatever," added, in the exercise of their discretion, "We believe that the instrument was skilfully used." The coroner afterwards expressed his concurrence in the verdict, and Miss Hall, as well as Miss Copeman, the nurse who was with her during the whole time of the injection, must be warmly congratulated upon the satisfactory manner in which they have emerged from a trying ordeal. Miss Denniston, superintendent of the Acland Nursing Home, was present during the proceedings, and gave conclusive evidence as to the training of Miss Hall, who has been a certificated nurse since 1892.

### A NURSE BURNT TO DEATH.

THE death of Miss Ellen Susan Cree, who had been for eighteen months a nurse at the London Homœpathic Hospital, Great Ormond Street, through burns, was enquired into by Dr. Danford Thomas at the Holborn Town Hall last week. The evidence showed that whilst combing her hair in front of the fire she set fire to herself, her flannelette nightdress being burnt completely away. A verdict of "Accidental death" was returned. The hospital staff was with Miss Cree almost instantly after the occurrence of the accident, and every means was at once taken to restore her. She died of shock on Friday, the accident having occurred on Tuesday. Miss Cree, who was only 25, was buried at Princetown.

### YORK HOME FOR NURSES.

THE principal feature at the annual meeting of the subscribers to the York Home for Nurses was the introduction, by Dr. Ramsay, of the scheme of enlargement. Owing to the great increase of work, the council have decided to build additional rooms, the first object being to provide separate accommodation for the five district nurses, and the next quarantine accommodation for nurses returning from infectious cases. It has also been determined to increase the number of beds for patients from 36 to 44, to provide a disinfecting chamber, and to add to the number of baths. There can be no question as to the need of these additions, and the sum of £2,000, the estimated cost, is all the more likely to be readily raised, because the council devoted a large portion of the increased income obtained from fees in 1900 to an augmentation of the nurses' salaries. The best way to secure a staff of good nurses is to treat them well, and while this is the policy pursued by the governing body of the York Home, its continued progress in its useful career may be regarded as assured.

### THE NURSES' ABSTINENCE LEAGUE.

A drawing-room meeting in aid of the Nurses' National Total Abstinence League was held on Friday at Stafford House, the Hon. Mrs. Eliot Yorke, the President, in the chair, and there was a considerable attendance of nurses. The Hon. Secretary, Miss Hilda Dillon, explained the origin of the League, which was formed in February 1897 in order to unite all nurses who were total abstainers, and to encourage them to enlist others in the temperance crusade. Mrs. Eliot Yorke referred to the important position which nurses occupy, and urged them to guard against the use of alcohol. Prebendary Barker, who was the principal speaker, also entreated nurses to have nothing to do with alcohol, and to use their influence in the interests of temperance. There are now 208 members of the League.

### GLASGOW TRAINING HOME FOR NURSES.

At the annual meeting of the subscribers to the Glasgow Training Home for Nurses the chairman, Sir Hector Cameron, referred to the loss the institution had sustained by the death of Lord and Lady Inverclyde, who had both taken a special interest in it. Lord Inverclyde, he added, had left £500 towards the scheme for the reconstruction of the building. This is now urgently needed. It is intimated that the cost of the building and furnishing will be £14,000, of which £6,000 had been obtained. The chairman admitted that the scheme was large, but contended that the expenditure was necessary if the institution were to keep pace with the times. He and his colleagues had, he said, "become conscious of the fact that the many improvements introduced by the progress of science in surgical treatment required so many changes in the way of appliances that the present buildings were quite insufficient for the needs of the institution, and under the circumstances they had determined to appeal to the public for funds to enable them to carry out their intentions." The citizens of Glasgow, we do not doubt, are sufficiently public-spirited to respond liberally to an appeal for such an excellent object.

### NURSING AT KILLARNEY.

The first annual meeting of the Killarney Nursing Association has just been held, and we are glad to learn that the results show, not only that the sick poor appreciate the services of a well-trained nurse, but also that there is every disposition to afford the requisite financial support. During the year 1900 the nurse attended 157 separate cases and paid 2,659 visits. As she nursed patients under the care of every doctor resident in Killarney, she evidently possesses the entire confidence of the medical profession. The total receipts were nearly £300, as against an expenditure of less than £94, and though the former included £192 collected by Lady Fitzgerald, for a fund to provide a Jubilee nurse in Kerry in commemoration of Queen Victoria, the excellent start made by the Killarney Association encourages the hope that it has come to stay, and to extend the scope of its operations as opportunity offers.

### THE NURSING HOME AT BISHOP AUCKLAND.

The annual meeting of the Bishop Auckland Nursing Association was held a few days ago. It appears from the annual report that the number of cases nursed during the year 1900 was 450, and the total number of visits paid 8,176. The receipts for the year amounted to £223 19s., a falling off of over £20 as compared with the previous year. The expenditure of £225 was less by £21 6s. Although the balance was practically the same, the financial condition was not considered satisfactory, and without the aid received from exceptional sources the needful funds would not have been raised. The Nurses' Home has been completed and furnished since the issue of the last report, and has been in occupation for six months. A considerable sum is required to defray the cost of the building and furnishing, and to meet all expenses.

### A TESTIMONIAL RETURNED.

The Newton Abbot Guardians decided after a long discussion to give a testimonial to Nurse Flaherty in accordance with the Visiting Committee's recommendation as follows:—

The Guardians of the Poor of the Newton Abbot Union hereby certify that Miss Cecilia Flaherty has been in their service as assistant nurse at the infirmary of the Workhouse from April 17th, 1899, to the present time, and that she has proved herself to be a very efficient nurse, and up to a recent date has carried out her duties to the satisfaction of the Guardians, Medical Officer, and Superintendent Nurse. During the past few weeks there had been friction amongst the nursing staff, and in consequence of a complaint from the Medical Officer the Guardians called upon Miss Flaherty to tender her resignation, which she did, and this was accompanied by the resignation of three other nurses. The Guardians, however, do not consider that this should militate in any way against Miss Flaherty receiving an appointment from any Board of Guardians who require the services of an efficient nurse.

We are not surprised to learn that Miss Flaherty has returned the "testimonial" to the Guardians, and prefers to rely upon that of Dr. Ley, who has seen her at work as a nurse.

### KIMBERLEY NURSING ASSOCIATION.

The second annual report of the Kimberley and District Nursing Association shows that the nurse, who carries on her increasing work in five parishes, paid 1,702 visits last year to 84 cases. Substantial financial support is given, there being a balance of more than £100 to the good. We infer that if a second nurse is required it would not be very difficult to provide her salary.

### SHORT ITEMS.

It has been decided by the General Committee of the Walsall and District Hospital that a nurses' home shall be proceeded with at once.—The next examination for the Certificate in Nursing and Attendance on the Insane will be held under the auspices of the Medico-Psychological Association of Great Britain and Ireland, on Monday, May 6th, 1901. Candidates should obtain a schedule from the registrar, Dr. Benham, City Asylum, Fishponds, Bristol.—Miss A. M. Browne, the new matron of the Birmingham and Midland Hospital for Skin Diseases, informs us that the particulars of her career were not correctly supplied to us. She received some training at the Royal Infirmary, Edinburgh, after which she was engaged in private nursing. She then went through the course of training at the London Hospital, where she was afterwards appointed sister, and obtained the certificate of training. Miss Browne holds the L.O.S. certificate.—Sisters K. McGonigal and L. B. Williams, of the Army Nursing Service, who are ill at Johannesburg, are reported "slightly improved."—The s.s. *Mohawk* arrived at Southampton from South Africa on Thursday last week; Sisters Castle and Griffiths were on board. On Friday, the *Canada* arrived, with Sisters Steele and Herbert, of the Cape Volunteer Nursing Service.—At Kent Assizes last week a labourer named Ernest Pring was sentenced to 15 months' hard labour for assaulting Miss Sarah Bryan, a nurse at the Sanatorium, Tunbridge Wells.

### Medical Lectures to Nurses.

By Carstairs Douglas, M.D., B.Sc. Edin., F.R.S., F.F.P.S. Glasgow, Professor of Medical Jurisprudence, Anderson's College Medical School, and Dispensary Physician, Samaritan Hospital, Glasgow.

### II.—HÆMORRHAGE FROM THE STOMACH AND OTHER INTERNAL ORGANS.

In our last lecture we considered the important question of hæmoptysis, or bleeding from the respiratory tract to-day we turn our attention to the stomach and other internal organs as sources of hæmorrhage.

Where the bleeding is from the stomach and the blood is got rid of mainly by the act of vomiting, the name "hæmatemesis" is employed. In many cases it is slight and does not cause much alarm, due largely to the fact that the blood has lain sufficiently long in the stomach, before ejection, to be acted on by the gastric juice, so that it assumes the appearance of coffee-grounds or hare-soup, and is not recognised by the patient as blood at all. In not a few cases of hæmatemesis the source of bleeding does not lie in the stomach, but is found in the respiratory tubes, the throat, or the nose, the blood in this case having been first swallowed and then vomited. The chief morbid conditions in which the hæmorrhage really takes place from the stomach itself are the following:—Gastric ulcer, cancer (or other new growths) of the stomach, advanced valvular disease of the heart, chronic lung disease, and cirrhosis of the liver (the bleeding here being consequent upon venous engorgement due to backward pressure); scorbutus, purpura, and other blood diseases; where corrosive poisons have been swallowed, and in the gastric attacks of that disease of the spinal cord called locomotor ataxia. In almost all these cases, although most of the blood may be vomited, a certain amount is apt to find its way into the small intestine, and the altered blood makes the motions black and often tarry-looking—the condition known as "melæna." Melæna is also met with in typhoid fever, in ulceration and tumours of the bowel, and in cirrhosis of the liver.

Of all the forms of hæmatemesis that due to gastric ulcer is the most important, as it alone is likely to prove fatal *per se*. A patient with ulcer of the stomach may die with comparative suddenness from one or other of two causes. In the first place the stomach wall may be perforated by the ulcer and a fatal peritonitis quickly ensue. Or, secondly, the ulcer may eat into the wall of a blood-vessel of some size, producing a sudden and violent hæmorrhage that may end the patient's life. This constitutes, indeed, an example of one of the gravest forms of internal bleeding that a nurse may meet with, and it is by no means a rare occurrence. In most of the cases the patient has already had some symptoms of gastric ulcer, perhaps being actually under treatment at the time. In another group there is a history of an old ulcer which is supposed to have been healed; while in yet a third class, there may be no symptoms (the ulcer is latent as it is termed) and without warning a serious, and it may be even a fatal, hæmorrhage takes place. In most cases the blood vomited is still florid, and may be mixed with food particles, mucus, &c. If the bleeding has been more gradual, the blood tends to be darker in hue, through partial conversion of hæmoglobin into hæmatin.

What can we do to favour the arrest of bleeding of so grave a nature? It is obvious that here, as in hæmorrhage from the lung, it is beyond our power to place a finger on the spouting vessel or otherwise directly control the flow. Here, again, we must trust largely to Nature to mend the broken wall, but we can do much to further her processes. In the first place the patient must be kept at absolute rest in a room which is cool, quiet, and secluded; bodily exertion of any kind must be strictly forbidden; and it is one of the nurse's chief duties to forestall all the patient's requirements and so reduce muscular movement to a minimum. In marked hæmatemesis feeding by the mouth should be entirely discarded and nutrition maintained by nutrient enemata and suppositories. Thirst is often much complained of, and for this a little ice may be allowed; but the patient usually desires more than is good for her, overloads the stomach with water, and may thus induce vomiting and bleeding again. It is better therefore to cut off nearly all water for drinking save a teaspoonful now and again; instead, the tongue and lips may be painted over frequently with a feather or brush dipped in glycerine and water (iced), and the hands, arms, and face frequently washed with almost cold water. Of all medicines opium is the most valuable in hæmatemesis; a grain may be given by the mouth, or a quarter of a grain of morphia injected under the skin. Indeed it may be said that absolute rest, rectal alimentation, and opium will control most cases of hæmatemesis. Of other useful medicines which act as hæmostatics, or arresters of bleeding, we may keep in mind ergot, administered best as ergotinine citrate $\frac{1}{100}$ gr. hypodermically; aromatic sulphuric acid, 5–20 ♏; lead and opium in 5 gr. pill; hazeline in drachm doses; and tannic acid 2–5 grs. at a time.

The bowels should be emptied every other day by a soap and water enema till the hæmorrhage has ceased for a week, when a dose of Carlsbad salts or other saline aperient may be given by the mouth. Another form of internal hæmorrhage is met with where the patient suffers from "hæmaturia," or the passage of blood in the urine. It occurs typically in calculus or stone, where there are malignant growths in the urinary tract, and in acute Bright's disease. It is rarely, however, so abundant as to endanger life, and a nurse's duty here lies chiefly in reporting the condition to the physician in charge: she is seldom called upon to treat it as an emergency.

One of the gravest forms of internal hæmorrhage is that occurring within or upon the brain: it is diagnosed, not by an external escape of blood, but by the serious symptoms which it produces. It will therefore be well to consider it in a subsequent lecture, which may be usefully devoted to a study of the various conditions which produce coma, convulsions, and paralysis.

### Where to Go.

Chelsea Town Hall.—Monday, March 18, 8.45 p.m. Lecture by Mr. Augustine Birrell, K.C., M.P.: "A Backward Glance at English Literature during the Last Century," in aid of the funds of the Cheyne Hospital for Sick and Incurable Children.

### To Nurses.

**We invite contributions from any of our readers, and shall be glad to pay for "Notes on News from the Nursing World," or for articles describing nursing experiences, or dealing with any nursing question from an original point of view. The minimum payment for contributions is 5s., but we welcome interesting contributions of a column, or a page, in length. It may be added that notices of entertainments, presentations, and deaths are not paid for, but, of course, we are always glad to receive them. All rejected manuscripts are returned in due course, and all payments for manuscripts used are made as early as possible after the beginning of each quarter.**

### Private Nursing in Durban and a Voyage on the "Canada."

By a Correspondent.

In the course of a chat with Sister Steele I gathered that the great need in Durban, as far as private nursing is concerned, is a central bureau for trained nurses.

### The Difficulty of Finding a Nurse.

"When I was about to take up army work," she said, "I had great difficulty in finding a substitute for the cases I was leaving in Durban. There were nurses to be had, I knew, but I did not know where to look for them. A central office, or, better still, a nursing institute like the V.N.I. at Capetown, would save doctors, patients, and nurses an enormous amount of running about, as well as delay."

"Have you any idea of the number of nurses doing private work in Durban?" I asked.

"I can only guess; but I should think there are from thirty to forty."

"What is the population of Durban?"

"At the last census the white population was taken at about 25,000; besides these there are now a great many refugees from the Transvaal—the town is too full of undesirables—and there are always Blacks, Hottentots, Coolies, Arabs, Chinese, and Malayans—a very mixed population indeed."

### Conditions of Private Nursing.

"Is it possible to make a good living as a private nurse?"

"The rate of pay is about the same as in England—three guineas a week for ordinary cases, such as enteric—but the work is very much harder. As a rule, one has to take the place of the mother if she is ill, and almost always this is the case in midwifery. There are the children and the meals to attend to, and often the actual cooking has to be done by the nurse. The black servants have very little sense of responsibility, and it is quicker and more satisfactory to do things oneself than to try and teach them."

"Does not the patient suffer from this arrangement?"

"We always explain that the mother and baby must be considered first, and that the rest of the family must do the best it can. But one has not the heart to refuse to do necessary things, though it frequently means night and day work. Some nurses do refuse to do the housekeeping, but I think they are none the better liked on that account. In England there is usually an aunt or some other relative who can come and see to the house, but in Natal young married people often have neither relatives nor friends near enough for this."

In spite of its disadvantages, however, Miss Steele likes the work, and quite intends going back to it when the war is ended and there is no more army nursing for volunteers. She first went out about thirteen years ago; and as her mother is engaged in the same profession, she may be said to be well acquainted with the conditions of private nursing in Natal.

### No "Little Comforts."

"The *Canada*," she said, "is the second vessel on which I have done duty since the war broke out. I was on the *Orcana* for some time. The *Canada* is a beautiful ship, almost as comfortable as a hospital ship, though she is only a transport. The ship's staff are exceedingly kind and thoughtful; and we had merely to say what we wanted for our men to have everything liberally supplied. But we suffered greatly from the lack of the tobacco, fans, &c., which were supplied during the early part of the time by the Absent-minded Beggar Fund and the Red Cross Society. As far as Durban, at any rate, is concerned, these funds are now exhausted; and some of the sisters have been wishing they could have been less lavish at first. Of course we were not allowed to save anything given for one voyage for another ship, but it is sad that the men are now entirely without the comforts they had earlier in the war. We felt this more especially in the tropics, for it was more than usually hot, and there was not a single fan."

"How many sisters were on duty?"

"Only two—Sister Herbert and myself."

"May I ask if you were trained in England?"

"Yes; at the Berks County Hospital, Reading; and I have been at Charing Cross and the Great Northern Central Hospital as well."

The *Canada* is sailing again for the Cape on Tuesday, and Sisters Steele and Herbert have asked to be sent back with her. With them goes also Sister Paterson.

### Nurses' New Quarters at the Royal Free Hospital.

The nurses' new rooms at the Royal Free Hospital were inspected by the governors and friends last week. It was a matter of disappointment that the formal opening by Princess Christian was necessarily abandoned on account of the Royal bereavements; but it is possible that something more festive may be done in the summer, when it is hoped that the other two floors will be finished. At present only the top floor is completed and occupied, but the addition is already proving of great advantage. There are two new wings, comprising nineteen rooms; and very bright and cheerful rooms they are. The prevailing colour is green, for the matron, who has had the responsibility of choosing, likes no colour so much as a real apple-green. The new rooms, which are at present occupied by the sisters and night nurses, extend to the right and left of the top landing; half the number face east and have an extensive view which takes in one of the Rowton lodging-houses and innumerable other buildings, stretching away towards Islington and Holloway and possibly Hampstead, while those on the other side of the corridor face the west and have a flood of afternoon sunshine—when it is to be had—as well as a view of the trees in the courtyard. They are cosy little rooms; one or two are oddly shaped and have taken the fancy of the sisters to whom they have been allotted; this is owing to the necessity of fitting a new storey on to an old building. They are all nicely furnished, partly by the matron and partly by the taste of the individual occupying them. The addition of a baize door to shut off the night nurses' rooms will be a great advantage and ensure the maximum of quiet during the day.

There is one little room which every nurse will find a decided blessing; this is a study, on which Miss Wedgwood has expended much thought, and which she has furnished herself. There is to be a fire all day in winter, and the room will be used by all the nursing staff "down to the youngest probationer" for reading, writing letters, notes of lectures, &c. It is a room for silence, for in the common sitting-rooms downstairs there is plenty of opportunity for social intercourse, and both the sisters' and nurses' sittingrooms have comfortable sofas and arm-chairs. Here all the chairs are upright, but low and comfortable; they were specially made by Messrs. Storey and Triggs, of green wood with rush seats. There is also a writing-table, given by Miss Wedgwood to the nurses some years ago, and the cushions and curtains ("cottage curtains," to fit the deep little window) are of material to match the green walls and chairs. It is rather a spartan room, as a study should be, but moreover comfortable, and certainly a room in which to work.

The most satisfactory result of the improvements is that the cubicle system will be superseded by a separate bedroom for every nurse. The cost of the building is defrayed out of the funds of the hospital.

### The Funeral Services for Queen Victoria.

By a Nurse at Pretoria.

On February 22nd the Holy Communion was the first of our Memorial Services, at which many were present. The other services attracted numbers of loyal and loving subjects.

The Cathedral was most tastefully draped in black and white. Facing the congregation on the front of the pulpit was an engraving of the Sovereign, in her robes of the first Jubilee celebrations, artistically framed in black, and relieved, as was also the whole of the pulpit, by broad white satin ribbon and silver initials of V.R.I. The choir stalls were draped in black, festooned with white ribbon, the altar rails in white with black trimmings, and the altar was black and white, with an occasional touch of purple, and very effective initials in frosted silver. Palms, with pots veiled with black and lightened by white ribbons, stood here and there, and on the lectern was a large wreath of white flowers carrying a silver V.R.I. across the space of the circle. Small purple and white crosses found places on the altar rails and at other points; the organ was draped in black and white, the whole chancel somehow suggesting hope as well as sorrow.

In the nave at the entrance to the two front pews hung the Royal Standard and Union Jack respectively—both shrouded in crape—and the two crosiers immediately behind were also wreathed in black and white. Long before the service began the Cathedral was packed to overflowing, none but British people, except a few foreign dignitaries in full dress uniform, being allowed entrance. The band of the Norfolk Regiment, with the big drum draped in crape, occupied part of the chancel, and played a beautiful funeral march by Beethoven as a voluntary. A few verses from the Burial Service were read and Psalms xxxix. and xl. sung. A short lesson, and then came the lovely hymn "Now the labourer's task is o'er." It seemed, indeed, as if we were committing a personal friend into the "Father's gracious keeping." Then followed an anthem which had been sung by Her Majesty's command at the inauguration of the national memorial to H.R.H. the late Prince Consort. The service was brought to a close by the band playing the "Dead March" in *Saul*.

In spite of the tremendous heat—quite unusual in intensity, we hear, for the time of year—the Cathedral was crowded to its utmost extent, some even standing at the door throughout the service. Lord Kitchener and his staff and crowds of officers were present. A guard of about 100 men, it seemed, sat in the body of the church, some sisters in full dress uniform affording the only piece of colour, with the exception of the gorgeous garments of the foreign dignitaries. On the following Sunday we sang "God Save the King" for the first time.

### Precepts for "Pros."

Dress and Manner.

Quiet and tidy must you be,
No noisy shoes should wear,
Your cap and apron white and clean,
And neat and smooth your hair.

The Proper Standpoint.

When first into the wards you go,
And see each suff'ring face,
As "human beings" think of each,
And not, as just "that case."

Beds.

In making beds, be sure you take
The clothes off, one by one,
Be quick, nor let your patient feel
He's chilled before you've done.

Washings.

The washings next claim all your thoughts,
Be sure the water's hot,
Nor take the patient's night shirt off
Till everything you've got.

Dusting.

Be thorough in your dusting, nurse,
No corner overlook;
Nor only dust where "Sister" sees—
The microbe loves a nook.

Food

Don't burn the patient's bread and milk,
Then say, it's very good,
Don't boil their eggs as hard as rocks,
Give appetizing food.

Feeding.

Don't pour the liquid down the throat
As if it were a sink.
With left hand, gently raise the head,
With right hand, give the drink.

Medicine.

Be sure you read directions first
Then there'll be no mistake,
And keep the label uppermost,
The bottle always shake.

Dressings.

Your hands you scrub and carbolize,
Forceps you must employ;
Don't keep the wound too long exposed,
Old dressings quick destroy.

Conclusion.

A conscientious nurse you'll make
If you these maxims mind,
And for your own this motto take,
"Obey, observe, be kind."

### Examination Questions for Nurses.

### INCIPIENT HIP DISEASE.

### RESULT OF FEBRUARY COMPETITION.

THE question was as follows: "What symptoms would lead you to suspect incipient hip disease in a child?"

### THE FIRST PRIZE.

"Nurse Edith" takes the first prize. Her paper is short and practical, and not overlaid with learned anatomical terms, often quite incorrect, and used apparently in some cases with the hope that the exhibition of so much "scientific" knowledge will veil the absence of practical acquaintance with the subject. She shows common-sense in the hint of the possibility of quite simple reasons for some suspicious symptoms, such as the presence of some slight local injury or an ill-fitting boot. This shows that she is accustomed to look at a subject all round before forming an opinion—a most important habit for a nurse.

### THE SECOND PRIZE.

"Nurse Victoria" is the second successful candidate. Her answer is almost identical with that of Nurse Edith, but it is more verbose, and she makes the mistake of mentioning a symptom (shrinking of the thick fleshy part of the leg) which does not occur in the first onset of the disease; it is a good answer, however, and well thought out.

### HONOURABLE MENTION.

"Shamrock," "Joe," and "Nurse Bébé" have all three gained honourable mention. I rather think this is the third time that "Shamrock" has been thus successful. I hope, therefore, we may soon see her in the first rank as a prize-winner.

### FIRST PRIZE.

The first symptom which would lead to a suspicion of the commencement of hip disease in a child would probably be slight lameness, unaccounted for by any wound of the foot or leg or by an uncomfortable boot. At the same time obscure pain, sometimes referred to the knee, may be complained of. The child would limp, having the limb slightly flexed and turned outwards and bearing upon the toes, and instinctively holding it stiff, and in such a position as to prevent the inflamed and painful surfaces of the joint from rubbing upon each other.

Another important symptom would be "night starts;" the child after falling asleep would wake with a sudden start and cry, caused by the jumping of the muscles and consequent pain by the jarring of the joint when the controlling power of the will is relaxed. Other symptoms, but which are not always present, are slight feverishness at night and loss of flesh and strength. NURSE EDITH.

### SECOND PRIZE.

The symptoms that would lead me to suspect incipient hip disease in a child would be:—

(i.) Lameness, which is almost always present, but varies in degree, and may only appear as a slight limp.

(ii.) Pain, sometimes severe, at others slight. It may be felt in the hip joint, but is more often "referred" to the knee, and occasionally to the inner part of the thigh and leg. If a child complains of pain in the knee, which, on examination, does not appear to be locally injured or inflamed, I should suspect the commencement of hip disease.

(iii.) Night-startings: the child waking up from its sleep with a violent start, as though frightened, and often giving shrill little cries or screams.

(iv.) Alteration in the position of the leg: the presence of the above symptoms would lead me to examine the child's leg and hip more closely, and I should place the child on his back, and compare the two limbs. The suspected leg will probably be found to be rotated outwards, and the thigh flexed on the pelvis; later this position is altered, the limb being rotated inwards.

(v.) Loss of movement in the hip joint, the child holding the joint rigidly, and there being pain and difficulty in flexing and extending the thigh, but more particularly in rotating it.

(vi.) Muscular wasting of limb: on comparing the two legs, I should probably find the suspected limb looking thin and shrunk by the side of the other, and on measuring the calves and thick part of the thighs I should find they were unequal.

(vii.) There may be heat and swelling round the joint, and the whole hip region may present a different outline to its fellow on the other side.

It is hardly probable that all the above symptoms would be present in one case, but loss or limitation of movement in the joint, pain, and lameness are rarely absent, and if combined with one or more of the other symptoms I have named, would lead me to strongly suspect the existence of incipient hip disease. NURSE VICTORIA.

### THE QUESTION FOR MARCH.

"What are the chief points to be attended to, and what dressings should you prefer to apply in a long case of extensive burn?"

### PREVIOUS QUESTION ON THE NEXT SUBJECT.

On March 10th last year there was a preliminary question as to the degrees of danger from various kinds of burns. It would be well for candidates to look this up. The present question does not touch on the subject of the dangers of burns, or the first aid to be given to the patient, but is intended to draw forth various views on the best treatment for the actual burns, supposing always that the doctor leaves the matter to the nurse as is often done, if she is a woman of experience.

### RULES.

The competition is open to all. Answers must not exceed 500 words, and be written on one side of the paper only. The pseudonym, as well as the proper name and address, must be written on the *same* paper, and *not on a separate sheet*. Papers may be sent in for fifteen days only from the day of the publication of the question. All illustrations strictly prohibited. Failure to comply with these rules will disqualify the candidate for competition. Prizes will be awarded for the two best answers. Papers to be sent to "The Editor," with "Examination" written on the left-hand corner of the envelope.

N.B.—The decision of the examiners is final, and no correspondence on the subject can be entertained.

In addition to two prizes honourable mention cards will be awarded to those who have sent in exceptionally good papers.

### Minor Appointments.

BIRMINGHAM AND MIDLAND EAR AND THROAT HOSPITAL. Miss Mary A. Brunton has been appointed sister. She was trained at the Parish Infirmary, Brownlow Hill, Liverpool.

### Death in our Ranks.

WE regret to record the death of Nurse Ina Cunninghame, of East Pilton Hospital, Leith, N.B., from enteric fever, contracted while in the discharge of her duties, on Wednesday, February 20th, 1901. Miss Cunninghame, who entered the above hospital in August, 1899, as a probationer, was only 23 years of age, and will be greatly missed by her fellow nurses and the sisters for whom she had worked.

WE also regret to learn of the death, at Chester Infirmary, from pneumonia, of Miss Blanche May Ramsey Williams, known professionally as Nurse Blanche. Miss Williams was 29 years of age.

### Echoes from the Outside World.

AN OPEN LETTER TO A HOSPITAL NURSE.

YOU will learn with interest that the committee appointed by the King recommend that a memorial to Queen Victoria be erected in the neighbourhood of the Abbey and Palace of Westminster or of Buckingham Palace, the memorial to include as its most prominent feature a statue of the Queen. The recommendation has been submitted to the King by Lord Esher, and a sub-committee has been appointed to consider the means which should be employed to carry this recommendation into effect. Subscriptions will be received by the Lord Mayor at the Mansion House and at the Bank of England. There is no doubt whatever that plenty of money will be forthcoming from the public, and that all classes will join in contributing to the national memorial of the most illustrious of English Sovereigns, though some little disappointment may be caused that it is not to assume the form of some large philanthropic enterprise for the benefit of humanity.

---

THERE will be rejoicing at the Antipodes that the Duke of Cornwall and York has been empowered by the King to confer the honour of knighthood on the occasion of His Royal Highness's mission to Australia. This means, of course that several public men in the Australasian and other colonies will receive the distinction at the hands of their Royal visitor, and some of the wives and daughters of well-known public personages across the seas will consequently be on the tip-toe of expectation and hope. I am afraid that the inevitable omissions may cause a few heart-burnings; but the Duke will, no doubt, be sparing, as well as judicious, in the exercise of his prerogative.

---

I DO not suppose that nurses took an absorbing interest in the elections for the London County Council on Saturday. But the event is one of great importance. Just before the poll closed I peeped into one of the Board Schools where the votes were being recorded, and I observed one lady exercising her rights as a ratepayer. A few women, I know, did nothing because they said they did not understand the subject, but some, possibly in accordance with a large proportion of the other sex, supported Progressive candidates with the idea of dealing a blow at the water companies. I doubt whether they desired to signify approval of a vast expenditure in municipal steamboats on the Thames and omnibuses on the streets, and it is to be hoped that any attempt on the part of the new County Council to house the working population will be protested against by all who do not wish to put an end to private enterprise. Miss Octavia Hill, who understands the question of housing of the poor better than most County Councillors, whether Progressive or Moderates, in an excellent review of the situation, which ought to have been published before the elections, says, "Withdraw the threat of municipal action, encourage the many agencies again to build, improve means of communication, lighten, if possible, the burden of rates, and the need of dwellings for the working population will, in my estimation, be gradually met in a satisfactory way, capable of variety and indefinite expansion." These seem to me to be the words of a wise woman which, however, will carry no weight with unwise men.

---

SENSATIONAL cases have, as rule, little attraction for me, but two which have been before us lately have such unusual side issues, that I feel I cannot leave them quite alone. The man Bennett, who was accused of murdering his wife on Yarmouth Beach, has been found guilty of the offence, and condemned to death. This was in spite of the evidence offered in support of an *alibi* by a witness who met Bennett, or someone resembling him, and of the same name, in the neighbourhood of Bexley, and walked with him for some time a few hours before the murder was committed. But the principal point of interest to the outside public is the question as to how much certain London halfpenny newspapers, by the articles which the Lord Chief Justice characterised as "monstrous and wicked," contributed towards the hanging of the man. The old ideas of Englishmen and Englishwomen that a man is innocent until he is proved guilty, have evidently become sentiments of the past, so far as those particular organs are concerned, and from the moment that the details of the outrage were reported, the contributors to this "gutter press"—as the brilliant council for the defence termed the offending journals—seem to have striven, especially by interviewing probable witnesses, to prove the man guilty. Those who cling to the method of trying a culprit, fairly and squarely, without prejudice, have one easy means of showing their disapproval of the more modern course, namely, by refusing to buy or to read papers which do not behave justly and honestly. But it is quite possible that the abuse which has been poured upon them from every side may produce a reformation. If so, the lesson will not have been in vain.

---

ANOTHER case of considerable interest is that of the girl of 22 years of age at Hampstead who, intending to destroy herself in her lover's presence, shot him dead instead. Conclusive evidence was forthcoming that she had no intention of committing murder, the accident having occurred owing to the effort on the part of the young man to save the girl from herself, and she was acquitted on the ground that the homicide was accidental. But to the suicide she pleaded guilty, and was awarded 15 months' imprisonment with hard labour. It must, I think, be admitted that the sentence is exceptionally severe, and though the practice of continually making petitions against sentences is much to be deprecated, I gather that a protest in the Eddington case would meet with support in several quarters. The girl has already suffered much in having killed the man she loved, and in having been kept in suspense for six weeks as to whether or not she would be convicted of murder. Now the heavy sentence which Mr. Justice Phillimore has seen fit to inflict, more especially the hard labour, for a young girl unaccustomed to such work, is likely to totally wreck her life and to render it impossible for her ever again to be a useful member of society.

---

NURSES who may be members of a temperance or a total abstinence league, as well as those who are not, will be interested in a decision given at the Lambeth Police Court the other day. A gentleman and his friend hired a hansom to drive to Farnborough in Kent and back again. On the way the fare treated the driver to five glasses of beer and one of spirits. The result, as might have been expected, was that the cabman was incapable of driving properly, upset the vehicle on the return journey, injured the man who hired him, and was fined next day for being drunk. Ultimately the injured man sued the cab proprietor for damages for personal injuries, but was unsuccessful because the jury agreed that treating the driver was "contributory negligence." If once the effects of such a verdict, and the risks which the public run by "treating" the drivers of public vehicles, are realised, they should go far towards stopping a practice which is reprehensible on every score.

### A Book and its Story.

### THE ANGLICAN CHURCH IN EGYPT.*

Mr. Montague Fowler's "Christian Egypt, Past, Present, and Future," has been written with the object of placing it within the reach of all classes of English-speaking people. He believes that among the increasing number of visitors who annually spend the winter on the Nile "there must be many who would gladly possess within reasonable compass an account of the work that is being done for Christ among the millions of Mohammedans who form nine-tenths of the population of the Nile Valley north of the Soudan." The author has had exceptional opportunities of verifying the facts which form the bulk of those chapters devoted to the work of the Christian Church in Egypt, as seen to-day in its various branches. The historical and statistical matter is a record of painstaking research and labour. His book will therefore be a valuable addition to the limited library of information on this deeply interesting subject. That the result of his labours may enlighten those who are as yet strangers to the religious question in Egypt, and be the cause of promoting the unity of "The Faith once delivered" among its followers in Christendom, is a wish which all earnest-minded readers will share with him. An invaluable index, carefully and intelligently compiled, makes access to any particular reference an easier matter than is sometimes the case, and a map of Khartoum showing the site of the Anglican church, with a striking portrait of Cyril, a Coptic Patriarch of Alexandria, gives an added interest to the contents. In writing of the three Christian churches in the East, established since primitive days, Mr. Fowler says, "Surely the story of the Coptic Church in Egypt, of the Nestorian Church in Assyria, and of the unhappy Armenian Church in Turkey, are amongst the many convincing proofs that we have of the fulfilment of Our Saviour's promise to His disciples, 'Lo, I am with you always, even unto the End of the World.' Without the indwelling of the Holy Ghost in their midst, these oppressed churches of the East must long ago have been swept away in the flood of Mohammedan conquest. But to their lasting honour they have chosen the better part, and in their loyal adherence to the Christian Faith have given thousands of their members to swell the noble army of martyrs whose blood is the seed of the Church." A short history of these churches is given, but the Coptic Church, which is the ancient Christian Church of Egypt, is the one for whose future the author has special concern. A considerable space is allotted to its history. The title is believed to have originated in a Greek word signifying Egypt, or to have come from that of a city, Coptos, lying 30 miles north of Thebes which, in past centuries of considerable importance, is now, under its present name of Kuft, but little known. The Coptic Church claims to be that branch of the Christian Church founded by the Evangelist St. Mark, A.D. 40. That after centuries of persecution and oppression at the hand of Mohammedan rulers, it should still possess followers to the number of over 500,000, is conclusive proof of its vitality, and it is remarkable that, in spite of the overpowering opposition they have met with, its followers still hold, almost intact, their ancient faith and Liturgy.

From the dawn of Christianity in Egypt, to its development and decadence during the first five centuries, Mr. Fowler passes on to the not least interesting portion of the book—the Mohammedan Conquest of Egypt. At that time the unhappy dissensions that arose in the Christian Church could not fail to have a weakening and disastrous effect on it, and prepare the way for the spread of Mohammedanism, "a creed that has for the past twelve centuries dominated a considerable portion of the world." It may not be out of place to quote for the benefit of readers to whom its tenets are not altogether known a passage bearing on the creed of Mahomet. "Born at Mecca in 569, he spoke with reverence of the Gospels: conceded that Jesus was a prophet and the greatest of all teachers sent from Heaven until, as he maintained, his own mission superseded that of Christ; that all honour and veneration was due to the Virgin Mary, whom he pronounced to be one of the four perfect women. He believed that Jesus Christ rose from the dead and ascended into Heaven; that He worked miracles. He looked forward to the second advent of Christ and to the Millennium. But he denied that Christ was crucified (affirming that a substitute was offered on the cross), and he refused to accept the divinity of the Lord, or to admit that the Mohammedans needed an atonement." It is not by the destruction of his faith that the Moslem can be won to accept Christianity, rather, the author rightly believes, it is to "dwell on all those details in which there is agreement; to enforce and enlarge those attributes of the Christ which he is taught to believe; and to encourage comparison between the Word of God and what 'the prophet' professed to have been a newer and special revelation to himself."

"Step by step the leaven of Christianity will do its work. Tenderly, and with every desire to avoid offence, the inconsistencies between Mahomet's teaching and practice may be pointed out. . . . By praising what is good in Mahommedanism and discounting those evils which were but selfish abuses of a nobler form of religion, having no part in the original scheme of Mahomet's system, the professor of Christianity will be able to draw a comparison between the teaching of Christ and that of Mahomet, to the advantage and superiority of the latter."

Mr. Fowler does not in any way attempt to deny what has been so often observed by travellers and others giving only casual attention to the fact, that the Coptic Christian of to-day does not compare favourably with his Mohammedan neighbour. This he considers is not so remarkable as the fact of the Coptic church having survived with so large a number of followers until the present day.

In the zealous, if not at all times discreet, efforts of Christians who from other churches and channels come forward to help "the down-trodden church of Egypt," a comparison in certain cases is irresistibly suggested to the author between them and the fable of the wolf and the lamb. "But where," he asks, "can strong, invigorating and disinterested help be found for those who cling to the priceless heritage—for those who have preserved it through centuries of persecution? . . . The answer can be given without hesitation. Such help can only be found among the true Catholic and apostolic branches of the Church of Christ in the Anglican communion. The British occupation of Egypt was not sought for, but was rather forced upon us by political and diplomatic necessity. It has opened out for the Anglican Church in Egypt an opportunity from which it would be little short of a crime to withdraw."

Bishop Blyth, as Bishop of Jerusalem, is largely responsible for the happy, fraternal and cordial relations existing between the authorities of the Coptic Church and our own. A new Anglican Bishop is shortly to be appointed for Egypt and the Soudan upon whom, under God, "a vast responsibility will be laid." "Christian Egypt" should be acceptable alike to the student and to the general public who are interested in the subject, which is treated with conscientious accuracy by an author who knows it thoroughly.

* "Christian Egypt." By the Rev. Montague Fowler, M.A. 1 vol (London: Church Newspaper Company.) Price 6s.

### Everybody's Opinion.

[Correspondence on all subjects is invited, but we cannot in any way be responsible for the opinions expressed by our correspondents. No communication can be entertained if the name and address of the correspondent is not given, as a guarantee of good faith but not necessarily for publication, or unless one side of the paper only is written on.]

### SHEFFIELD ROYAL HOSPITAL.

"THE MATRON" of the Sheffield Royal Hospital writes: With reference to a statement under "Notes and Queries" in your last issue, may I say that in the Sheffield Royal Hospital probationers are certificated at the end of two years, but if mutually desired training may continue another year, at the end of which time a three years' certificate is given?

### A WARNING.

"THE MATRON of the General Hospital, Great Yarmouth," writes: With reference to the letters in your last two issues, I beg to state that a man who said his name was Alfred Morris, and his age 23, was admitted into this hospital on February 8th, 1900, under exactly similar circumstances to those described. It would be interesting to learn how often the apparent fraud has been perpetrated.

### RESPECTING A MINOR APPOINTMENT.

"THE MATRON of Saint George's Union Infirmary, Fulham Road, S.W.," writes: I wish to point out that there is an error in an announcement in the "Minor Appointments" column of the last number of the NURSING MIRROR. It is stated that Nurse W. Carpenter was trained at this infirmary. This is not exact, as Nurse Carpenter did not remain long enough to qualify her to sit for examination, as she left after one year and eleven months' service. She was therefore not "trained" here in the usual meaning of the term.

### NURSES' HOMES.

"A PENSION FUND NURSE" writes: With other nurses I have for years felt the want of "nurses' homes," like those already existing for governesses, where we could enjoy the comforts and advantages of living for a small charge in any city or town of importance. I have had rooms in a suburb of Manchester, and I shall want others when I leave my present nursing. I am trying to put by money for old age and I do not want to spend it in expensive boarding. Perhaps you can see your way to take up the matter.

### NURSING IN PRISONS.

"THE WARDEN OF THE ROYAL VICTORIA HOMES, BRENTRY, NEAR BRISTOL," writes: Some months ago I remember a number of nurses expressed in your columns a wish to enter the prison service so that they might help those who had become inmates of such establishments. Should this letter meet the eye of any of those ladies, may I suggest that she refers to your advertisement columns? The managers of these Homes are increasing their staff, and although in our case stone walls do not a prison make, yet these are the cases needing attention under conditions which in every way are more hopeful than those existing at the establishments referred to.

### NURSES AND THE INDUSTRIAL LAWS.

"MRS. H. J. TENNANT" writes: I should be grateful if you would correct two errors which I notice in the very admirable summary you were good enough to publish of my lecture. The first is that 400 cubic feet of space is required during overtime only where artificial light is used. It is required during *any period* of overtime; and the Secretary of State may by order . . . modify this proportion for any period during which artificial light, other than electric light, is employed for illuminating purposes. The second—a slight error—is in reference to the Truck Acts. The *earlier* Truck Acts which prohibit payment except in the current coin of the realm, are broken less in London than in the country. Payment in goods is more commonly the offence of the small country tradesman who is agent for a large firm. But the Truck act of '96, which deals with fines and deductions for materials or damaged goods, is as freely broken in London, I think, as in any other part of the country.

### WORKHOUSE VISITING.

MISS LOUISE TWINING writes: It is somewhat surprising to me to see this subject now being discussed as a new suggestion when it is nearly fifty years ago that I first was convinced of its necessity, both for the welfare of the inmates and to make these then secluded institutions and their condition known to the outside world. This conviction led to the formation of the "Workhouse Visiting Society" in 1858, at the meeting of the Social Science Association at Liverpool, under the sanction and patronage of some of the highest authorities in Church and State. Its work was continued, and a journal published for some years, till it was believed that its objects were accomplished and visitors were nearly everywhere admitted. But we felt it was absolutely essential to success that organisation must exist and rules be formed, or confusion would certainly ensue. I may mention that a pamphlet on "The Duty of Workhouse Visiting," issued many years ago, may be obtained from our Secretary of the Women Guardians' Society, 4 Sanctuary, Westminster.

### TWO CONCEPTIONS OF NURSING.

"ALPHA" writes: "A Matron of Experience" lays herself open to criticism, and shows her lack of sympathy as a true woman (to quote her own words) when she allows herself to write of a nun as "an unsexed and unsympathetic nonentity" incapable of making a sensible, sensitive, or sympathetic nurse. What true woman would thus write of a band of heroic ladies, chiefly of good birth, who in all our great cities, and in South Africa, are daily seeking to alleviate the sufferings of God's creatures without expecting any remuneration in this life? Is it also just to condemn a patient as unsafe for any true woman to nurse who, possibly for want of thought or inexperience, and not from any motive of insult or disrespect, suggested his nurse dining with the housekeeper in his hotel, whereas had the nurse in refusing exercised a little tact, instead of immediately leaving, the difficulty might have been overcome? No woman should take up nursing, or will ever make a good nurse, whether trained or not, whose first consideration is her own comfort and social status, thereby causing her patient unnecessary pain and discomfort, and losing the respect which is every nurse's due.

"ANOTHER MATRON" writes: The letter of "A Matron of Experience" is of a somewhat startling nature. I am especially surprised at her experience of clergymen, as my feeling is that as a rule they are rather prone to idealise the nursing profession. Certainly I have yet to meet the clergyman who does not treat me as at least his social equal, and when I do meet him I shall not trouble him with my own further acquaintance. As for the much vexed question of private nurses and their meals, shall we ever be any nearer the solution of the problem? I most thoroughly sympathise with any woman of refinement who may be subject to such an annoyance as to be expected to put herself on terms of equality with those whose education and manners are inferior to her own; but as it is impossible to lay down the law in other people's houses, I fear that, except for those nurses who are attached to institutions whose rules protect them from such indignities, things must be taken as they come. We must not forget that when there is sickness in the house it generally means more or less complete disorganisation of the household, extra work for the servants, and much anxiety for the mistress, and these reasons may be some excuse for the nurse being inconvenienced.

A. F. C. writes: May I remark that anyone who allows herself to speak of a nun as an "unsexed nonentity" is not only hopelessly blinded by prejudice, but is extremely rude? There are in the nursing profession many nuns, and it is always rude and the sign of a weak cause to make use of terms which one knows will be objectionable. Perhaps the nurse who left her patient at the hotel was a lady but had she happened to remember that her patient was the first consideration she would doubtless in a very short time have made her own arrangements as to how and when and where she would take her meals, if she objected to take them with the housekeeper, who, it may be, was as good as herself. Naturally we would all prefer to take our meals alone; but in private nursing this is not always convenient. It seems a pity that anyone aspiring to such a high degree of nicety should have deigned to join a profession in which one is obliged to do many things that one does not appreciate. But having done so she had better learn that as long as she can get all that she requires for her patients she is herself of minor importance, and not *vice versâ*. As to the spirit that "A Matron of Experience" talks about, I have never seen it shown towards nurses, and she is wrong to generalise from a few incidents. She will do well to cultivate a little more kindliness, or else keep her opinions to herself and try not to be more aggressive than is necessary. In this century, surely, such narrow minded bigotry is be out of date.

### NURSES AND THEIR MEALS.

"USED UP" writes under this head: Before people condemn the nurse for daring to rebel at her food, accommodation, &c., let them be quite sure that they understand all that she has to go through whilst nursing a private case. To begin with, it is often her fate to spend twenty-two hours out of the twenty-four in the patient's room, in a more or less tainted atmosphere; for in some cases no amount of fresh air or disinfectant will banish the fœtor—the patient is often afraid of the first and objects to the second. Sleep is most irregular, frequently the only arrangement being that instrument of torture, a chair-bed in the sick-room. I have for weeks together only got four or five hours' sleep in the twenty-four. Nobody who has not tried it can understand the awful brain fatigue and the feeling that one would give one's very soul for a quiet night in bed in a wholesome room; but the nurse is expected to be cheerful, patient, full of tact and resource, to bear with the fads and vagaries of both patient and friends, however much her nerves may be racked from want of sleep, to be accurate and collected at the doctor's visit even though her brain be reeling. Meals are often hurried and irregular, sometimes not eatable. Coming straight from a sick-room scene, eyes, nose, and throat full of the horror (nurses will know what I mean) it is not easy to tackle any meal, however tempting, but when it consists of cold, overdrawn tea, bread, butter, meat, or egg, all sour or tainted, eating is impossible. This has been my lot at several houses, and in every instance but one the people were wealthy. I have never complained, but I imagine if I had done so, a certain section of the public would have condemned me as greedy and self-seeking. The food was such as I have described at my first case. I had no money to spend on supplementary food, having only just begun to earn my own living, and dared not risk my reputation by grumbling, so I just starved. It took many months to recover from this case. I shall doubtless be told that I have mistaken my vocation, and that a true nurse will not mind any hardship. On the contrary, I love the work, and have stuck to it in spite of several breakdowns caused by lack of sleep and good food. I only want fair play, and think that eight hours' uninterrupted rest should be made compulsory. As long as nurses are left to the mercies of their employers they will continue to get old and tired before their time, or, what is worse, irritable and "cranky."

### Presentations.

CHELSEA INFIRMARY.—On Thursday last week the assistant matron of Chelsea Institution was presented with a handsome travelling clock bearing the inscription, "Presented to Sister Mackay from the Nursing Staff of Chelsea Infirmary, 1901." Also a leather writing case, with her monogram in silver, and a silver penholder, accompanied by a letter of good wishes signed by each member of the nursing staff. Miss Mackay has spent several years in the infirmary, and her genial and kind-hearted disposition has made her beloved by everyone who has had the privilege of working with her. In losing her the staff feel that they part with a true friend, who ever had the welfare of those under her at heart, and it is with the utmost regret that they bid her farewell.

### The Hostel of St. Luke.

### ANNUAL MEETING.

THERE was not a great deal to interest nurses in the meeting at Church House, Westminster, on Thursday. The speakers dealt with the need of such an institution as that at 16 Nottingham Place on account of the serious and increasing poverty of the clergy. St. Luke's Hostel was founded seven years ago to bring the best surgical care and skilled nursing within the reach of the clergy and their families. At that time there were difficulties in admitting persons of moderate means to the public wards of the great hospitals; moreover, country medical men were unable to keep abreast of new discoveries and appliances, and the Rev. Dr. Wace quoted a case in which an entirely false diagnosis had been made from this cause. A clergyman's wife was told by her own doctor, and also by the medical man in the nearest county town, that a serious and possibly fatal operation would be necessary. She was fortunate enough to be admitted to a London hospital, where within a few hours, she was informed that no operation whatever was needed, and that she might go home with every prospect of recovery.

The Bishop of Rochester, who kept his appointment as chairman, notwithstanding almost complete loss of voice, was unable to do more than assure the meeting of his sympathy with the work of the institution; and the more formal business was kindly transacted for him by Dr. Wace. This consisted of the adoption of the annual report; the appointment of officers for the year 1901; some alterations in the constitution; a resolution urging the need of new premises; and thanks to the members of the medical staff.

The report stated that 87 patients had been treated in 1900, as against 92 in the previous year. The difference arose from the conclusion at which the committee had arrived, with great regret but without hesitation, that to ensure the greatest efficiency it was impossible to accommodate more than seven patients at one time in the present building. The cases had included many of a very serious nature, thus showing that the Hostel had gained and was retaining the confidence of the profession and of the public.

The Rev. Duncan Travers, representing the Universities' Mission to Central Africa, spoke of the boon the Hostel had proved to many mission clergy. The work of the nurses had come under his own observation when visiting patients connected with the mission.

Mr. Pickering Pick replied to the vote of thanks to the medical staff. His warmest sympathy centred in the work of the institution, but (it was a very large But) their hands were tied, and their efforts were seriously handicapped, by the place in which they had to work. A private house could never be successfully transformed into a hospital; the construction, sanitation, ventilation, &c., were quite different in the two classes of buildings. The house in Nottingham Place was most seriously ill adapted for the purpose, and entailed work on the nursing staff which would not otherwise be required. Speaking of the nursing staff, he said he should like to record the appreciation by the medical staff of the way in which the nurses carried out all their instructions, and rendered them invaluable help. "My feelings," said Mr. Pick, "have carried me away: I have gone off from the resolution. But out of the abundance of the heart the mouth speaketh."

### Appointments.

WETHERBY ISOLATION HOSPITAL, SICKLINGHALL.—Miss Blanche Drake has been appointed nurse matron. She was trained at Essex and Colchester Hospital, and has since been, for five years, on the staff at the York Home for Nurses.

BRADFORD CHILDREN'S HOSPITAL.—Miss Flora J. B. Cameron has been appointed matron. She was trained at the Western Infirmary, Glasgow, and has since been night superintendent, house sister, and assistant to the lady superintendent at Bradford Royal Infirmary.

### For Reading to the Sick.

### THE DISCIPLINE OF SUFFERING.

ANGEL of Patience! sent to calm
Our feverish brows with cooling palm;
To lay the storms of hope and fear,
And reconcile life's smile and tear;
The throbs of wounded pride to still,
And make our own our Father's Will!

There's quiet in that Angel's glance,
There's rest in his still countenance!
He mocks no grief with idle cheer,
Nor wounds with words the mourner's ear;
But ills and woes he may not cure
He kindly trains us to endure.

O thou who mournest on thy way,
With longings for the close of day;
He walks with thee, that Angel kind,
And gently whispers, "Be resigned:
Bear up, bear on, the end shall tell
The dear Lord ord'reth all things well!"

—*J. G. Whittier (adapted).*

"Ye have need of patience, that, after ye have done the Will of God, ye might receive the promise," says St. Paul; and our Saviour said, "In your patience possess ye your souls." The greatest happiness of anyone is "to possess his soul;" and the more perfect our patience, the more fully we do so possess our souls. Call often to mind that our Saviour redeemed us by bearing our sins and suffering for us, and in like manner we must seek our own salvation amid sufferings and afflictions; bearing our troubles with all the gentleness we can possibly command. Do not limit your patience to this or that kind of trial, but extend it universally to whatever God may send, or allow to befall you. Obey your physician, and take all medicines, remedies, and nourishment for the Love of God, remembering the vinegar and gall He tasted for love of us; desire your recovery that you may serve Him; do not shrink from languor and weakness out of obedience to Him; and be ready to die if He wills it, to His Glory, and that you may enter into His Presence. The bee while making its honey lives upon a bitter food. And just as the best honey is that made from thyme, a small and bitter herb, so that virtue which is practised amid bitterness and lowly sorrow is the best of all virtues. Gaze often inwardly upon Jesus Christ crucified, and remember that none of your sufferings can ever be compared to His, either in kind or degree, and that you can never suffer anything for Him worthy to be weighed against what He has borne for you. Offer all your pains and weakness to our Dear Lord, and ask Him to unite them to His sufferings.—*F. de Sales.*

Called aside,—
From the glad working of thy busy life,
From the world's ceaseless stir of care and strife,
Into the shade and stillness, by Thy Heavenly Guide,
For a brief space thou hast been called aside.

Lonely hours
Thou hast spent, weary on a couch of pain,
Watching the golden sunshine, and the falling rain;
Hours, whose sad length only to Him was known,
Who trod a sadder pathway, dark and lone.

Laid aside;—
May not the little cup of suffering be
A lovely one of blessing given to thee?
The cross of chastening sent thee from above
By Him Who bore the Cross, Whose name is Love.

*Anon.*

### Notes and Queries.

**The Editor is always willing to answer in this column, without any fee, all reasonable questions, as soon as possible.**
**But the following rules must be carefully observed:—**
1. **Every communication must be accompanied by the name and address of the writer.**
2. **The question must always bear upon nursing, directly or indirectly.**

**If an answer is required by letter a fee of half-a-crown must be enclosed with the note containing the inquiry.**

*Red Cross.*

(247) Can any trained nurse wear the badge of a Red Cross added to her uniform, or does it belong to some particular hospital or community?—*Doubtful.*

The Red Cross is the symbol worn by those engaged in attendance on sick and wounded on the battlefield, which, by virtue of the Convention of Geneva, entitles them to protection from both combatants. It is also a Royal decoration.

*Surgical Training.*

(248) What are the duties of a nurse as regards a still-born child? Is she supposed to arrange with the undertaker as well as to prepare the child's body for burial? 2. Is there a surgical hospital where I could get a few week's theatre training by paying for it? I am a trained nurse, but lack experience in this particular.—*Nurse W.*

1. A nurse's duties end with her services to patient and child. The head of the family should arrange for the funeral. 2. You would probably get what you seek by advertisement.

*Face Massage.*

(249) Could you kindly tell me where lessons are given in face massage?—*E. G. D.*

This is hardly within the province of nursing, but the Society of Trained Masseuses, Buckingham Street, might assist you.

*Meat Press.*

(250) Will you kindly tell me who are the makers of the special press for extracting meat juice mentioned in the NURSING MIRROR for November 17th?—*A. S. B.*

Messrs. R. and W. Wilson and Son, 90 Wardour Street, W.

*Probationer.*

(251) I shall be eighteen in March. What is the earliest age at which I could become a probationer?—*B. L.*

In a few children's hospitals at twenty, but in most general hospitals not until you are twenty-three.

*Leeds.*

(252) Can you give me the address of an institution in Leeds which is worked on the co-operative system?—*Rose J.*

The Leeds Trained Nurses' Institute, 21 Hyde Terrace, is the only association in Leeds for general nursing managed by a committee.

*Imbecile.*

(253) Can you tell me where a girl of thirteen, a dumb imbecile, could be received? Her father could pay 4s. or 5s. a week.—*W. E. A.*

Write to the Secretary, Earlswood Asylum for Idiots and Imbeciles, 36 King William Street, London, E.C. The asylum is at Redhill. Also to the Medical Superintendent, Darenth Schools, near Dartford, Kent.

*Preparing Pupils.*

(254) I am a trained nurse holding a three-year certificate in general nursing, and that of Glasgow Maternity Hospital for midwifery. Am I (not holding the L.O.S. certificate myself) qualified to train a pupil for the L.O.S. examination? If so, what fee is it usual to charge for instruction?—*Enquirer.*

Certainly. Write for syllabus of necessary subjects and regulations to the Secretary, London Obstetrical Society, 20 Hanover Square, W. The usual fee, without board-residence, is from £7 5s. for full course of instruction.

*Training School.*

(256) Is the Central London Sick Asylum a good training school for nurses? What kind of cases are taken there, and why is it so little known?—*A. S. D.*

It is a large school of 264 beds, preparing probationers for a three-year certificate in general nursing. See nursing guide books.

*Maternity Cases.*

(256) Are there any institutions which employ certificated maternity nurses who have no general training? 2. What is the best way of getting private cases?—*Miss K.*

1. See our advertisement columns. 2. Introduction through friends and medical men.

*Patient.*

(257) Will you kindly tell me how to gain admittance as a patient into the London Hospital, either free or by paying a small sum?—*Ignoramus.*

Apply to the secretary.

*Phthisis.*

(258) Can you tell me of a home where a young footman in the early stages of phthisis could be received for the open-air treatment?—*Nurse A.*

The North London Hospital for Consumption and Diseases of the Chest, Mount Vernon, Hampstead, N.W., would probably suit the case.

**Standard Books of Reference.**

**"The Nursing Profession: How and Where to Train." 2s. net; post free 2s. 4d.**
**"The Nurses' Dictionary of Medical Terms." 2s.**
**"Burdett's Series of Nursing Text-Books." 1s. each.**
**"A Handbook for Nurses." (Illustrated.) 5s.**
**"Nursing: Its Theory and Practice." New Edition. 3s. 6d.**
**"Helps in Sickness and to Health." Fifteenth Thousand.**
**"The Physiological Feeding of Infants." 1s.**
**"The Physiological Nursery Chart." 1s.; post free, 1s. 3d.**
**"Hospital Expenditure: The Commissariat." 2s. 6d.**
**All these are published by THE SCIENTIFIC PRESS, Ltd., and may be obtained through any bookseller or direct from the publishers, 28 and 29 Southampton Street, London, W.C.**

